# An empirical study of Chinese students' behavioral intentions to adopt 5G for smart-learning in Covid-19

**DOI:** 10.1186/s40561-021-00172-9

**Published:** 2021-10-29

**Authors:** Sayed Kifayat Shah, Zhongjun Tang, Sayed Muhammad Fawad Sharif, Arifa Tanveer

**Affiliations:** 1 Present Address: College of Economics and Management, Beijing University of Technology, Beijing, China; 2 Present Address: School of Management, Northwestern Polytechnical University, Xi’an, China

**Keywords:** Covid-19 pandemic, Smart-learning, Technology acceptance model (TAM), Social Practice Theory (SPT), Consumer adoption intentions

## Abstract

The social distancing due to the Covid-19 epidemic has disturbed all sectors of society, including education. To maintain normal operations, it is necessary to adapt quickly to this situation. Many technologies and platforms have rushed to offer their support to users. This article adopts a critical perspective to reflect on the factors that may cause the hasty adoption of 5G smart learning technology. To investigate students' intentions toward smart learning, this article provides a theoretical framework premised on the technology acceptance model (TAM) by adding components from the social practise theory (SPT). Based on data analysis through Structural equation Modeling (SEM) of a survey (n = 375) conducted in China, we found that the choice of 5G smart-learning technology depends on the combined effect of Material (MAA), Meanings (MEA), and Competency access (COA) factors. The results illustrate that these are the effective factors for student’s intentions to adopt 5G smart-learning technology. These outcomes are intended to aid service providers and decision-makers in developing effective ways to increase smart learning use. These findings can also enable us to identify challenges affecting smart learning adoption and to contribute to the design and proper supply of smart learning programs in other countries.

## Introduction

The coronavirus disease (Covid-19) pandemic showed disrespect for human-made borders, and it took only a few months to stop the world, demonstrating our close ties with the people on earth. Many countries closed educational institutions across the country to prevent the virus's spread. It has led to an unprecedented scale of distance education testing. In this sense, the need and use of advanced technology for education quality have significantly increased. Therefore, it has been found essential to utilize and implement the advanced technologies that constitute this new changing environment. Compared with traditional learning, the concept of smart-learning has made more incredible progress due to location flexibility, timely application, cost-saving and many other benefits. We believe that the availability of 5G technology will further strengthen smart learning. It will help to overcome time and space constraints and will achieve fast communication, best teaching content, and high-speed networks. As educational institutions are also experiencing essential changes, and their behavior is like that of large companies (Rossi, 2014; Sinclair et al., 2021). It has three main stakeholders: employees, students (customers), and society. Without centring on any of these stakeholders, the institution will not be able to sustain itself. In this changing environment, students (customers) early adoption of 5G technology in educational institutions will become a competitive advantage to attract and retain new customers.

What is the customer's intention to adopt 5G? What factors will affect the student's adoption of 5G technology for smart learning? Reviewing the previous research, the main purpose of this article is to establish an inclusive model to consider the critical and influential factors. As widespread and concrete research for accepting smart-learning through 5G has not been done yet. Therefore, this study will be beneficial for developers and researchers in further developing smart learning research. Further, previous studies haven't yet provided any accurate and comprehensive classification for the factors of adoption in the context of smart learning. This study divided these factors into three categories: Materials, competencies, and Meanings. This classification will help developers to make enhanced decisions when prioritizing and planning smart learning implementation issues.

The rest of the paper's work bestows the literature and theoretical background followed by the proposed model and hypothesis. The methodology part is observing the data followed by the analysis and results. The study concludes by discussing the results and contributions, along with few limitations and future considerations.

## Theoretical background

### The Covid-19, a push to smart learning

At present, the digital revolution and virtualization have dramatically altered all economic, social, and formal education systems (Lorenzo & Gallon, 2019; Winthrop et al., 2016). We have also discovered and intensely studied technologies used to help students learning. Today, advanced technology is used as a means or tool to access learning content (Daniel, 2012), research, construction, communication (Bruce & Levin, 1997), assessment (Meyer & Latham, 2008) and expression (Goodman, 2003). With the mobile and advance internet technology, mobile learning has become an essential mode of learning. Compared with traditional static education, mobile learning emphasizes students' mobility. Further, the support of ubiquitous technology has brought about other changes, changing the way of learning from mobile to ubiquitous, emphasizing that learning can be carried out anywhere, regardless of time, location, or environment (Hwang et al., 2008). Today, Smart learning is a novel model of using IT in educational institutions. The global covid-19 has instigated large-scale behavioral and institutional shock effects in almost all parts of life. The influence of this epidemic on learners is extraordinary (Chang et al., 2021). It restricted more than 150 million students worldwide to their homes (Teräs et al., 2020). Due to large-scale shutdowns, affected countries have been enforced to seek out speedy solutions and to shift to online learning (Jandrić 2020; Presti et al., 2021). The prompt change from classroom teaching to online has left behind deeper insights related to national education policy, theoretical basis and premise (Sunarto, 2021). This new environment enabled students to use advanced technology and techniques of learning (Cheung, 2014; Fayez et al., 2021; Marinova et al., 2017). Researchers and educators argue that this concept (smart learning) should not be restricted to only smart devices use. This kind of environment is a digital environment or virtual place, through a structured approach or a self-regulated process, fully supervised or semi-supervised to learning and teaching (Cook, 2010). With the increase in technical complexity, the concept of smart learning is evolving into a contextualized and virtualized ubiquitous environment. The modern concept of smart learning has become so clear that the limits between informal and formal learning have almost disappeared (Annoni & Kozovska, 2010). Smart institutions are converting into social and personal centers of learning, where teachers, students, and entire communities can take part in activities (Lorenzo & Gallon, 2019). Hwang (2014) and Scott and Benlamri (2010) believe that smart learning is ubiquitous and context related technique. Gwak (2010) suggested the idea of smart learning as it has more content and learners than device-oriented learners and it is efficient, intelligent, and personalized learning. Kim et al., (2012) believe that this type of learning is service-oriented and learner-centered, rather than using the equipment. Middleton (2015) also illustrates learner-centred smart learning and how they can benefit from smart technology. According to the Korean Ministry of Education, Science and Technology Smart Learning is as follows:

S: Self Directed: It means that the system of education moves towards a system of self-learning more than ever before. The role of students changes from adopters to creators of knowledge.

M: Motivated: It refers that education seeks creative problem solving and personalized assessment while being mindful of the experience.

A: Adaptability: Adaptability means increasing the flexibility of the system of education and adapting learning to personal preferences for future careers.

R: Resource enriched: Resource enriched says that smart-learning uses rich content for both the public and private sectors.

T: Technology embedded: Technology embedded means that students can learn anytime and anywhere through advanced technology in the education environment.

The government, academic, and industrial researchers should find ways to integrate existing technologies into 5G technology. They should also find possible ways to build an adoption environment as this will overcome all the limitations in the existing technologies.

### Previous work

Learning through advanced technology provides a platform for students to learn regardless of their location (Khan et al., 2019). Some empirical research has discovered how such type of learning affects pedagogical tactics, endorsing that new pedagogical approaches in education could help students learn (Khan et al., 2019). According to growing studies, internet technology and mobile devices are increasingly being used to assist students all around the world (Churchill et al., 2016). Several studies (Alharbi et al., 2017; Khan et al., 2019) assert that learning assisted by advanced technology has worked as a significant accelerant in education systems, where the evolution of new learning approaches has profited from a momentous evolution that boosts the learning efficiency of both educators and students. The integration of internet and mobile technologies in education appears to have yielded positive results. Global revenue through E-learning is predicted to reach $65.41 billion by 2023. However, as a result of the covid-19 epidemic and the evolution of the 5G internet technology, education has fundamentally changed its essence. When using the smart learning concept, it is beneficial to undertake a more detailed study to finalize various learning strategies (Putnik, 2016). Before applying smart learning in education, student’s perceptions about the technology should be explored. The majority of past research has concentrated on students' adoption of learning through technology, with intent as a dependent variable. Using the Unified Theory of Acceptance of Technology (UTAT), (Abu-Al-Aish & Love, 2013; Venkatesh et al., 2003) discovered that effort and performance expectancy, quality of service and personal innovativeness affect mobile learning acceptance. When Briz-Ponce et al., (2017) evaluated the variables of learning technology acceptance using TAM, they discovered that perceived ease of use and perceived usefulness influenced the adoption of such technology. Lin et al., (2020) explored students learning intention using TAM ∪ TPB models. Smart learning, on the other hand, is a brand-new approach to education that has emerged in the previous decade and got importance, particularly after Covid-19. As a result, we anticipate that users will assent or reject smart learning based on their willingness to adopt smart learning technologies. Therefore, our study extended the traditional TAM by incorporating three new factors from social practice theory (SPT). Although there have been several studies in this field, research findings of student’s adoption of smart learning in covid-19 are still few. Research on Chinese students’ intentions of smart learning is a gap that needs to be filled.

## Proposed model and hypothesis development

**Fig. 1:**
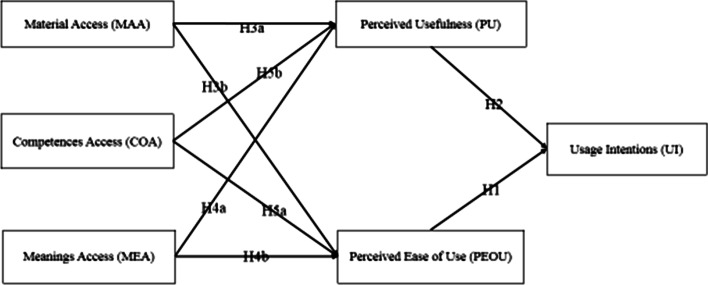
Proposed model

The technology adoption literature offers a variety of models and theories that explain the process of technology adoption. The most obvious of these is the TAM proposed by Davis (1993). Many researchers have confirmed the reliability and robustness of TAM in predicting and interpreting technology acceptance behavior (Venkatesh et al., 2003; Hasan, 2007; Kim et al., 2016). TAM is considered to be a universal model used to solve consumer acceptance of innovative technologies (Cheung & Vogel, 2013; Ha & Stoel, 2009; Ullah et al., 2021). Previous studies have also explored and validated its feasibility and usefulness (Cheng, 2012; Ha & Lee, 2019; Hubert et al., 2017). However, its basic structure ignores some important aspects, which is one of the reasons for criticism and extra expansion. Davis (1993) also suggested adding other factors to the traditional TAM based on the study's background. Taking into account the innovative characteristics of 5G technology, in this study, we used the TAM as the elementary theoretic model to know the experiences of consumers' willingness to practice 5G technology for smart learning. The factors related to Social practice theory (SPT) have never been addressed before. The SPT can interact with the concepts of social psychology, which may provide useful insights that can theorize and change the practice of social significance meaning (Kurz et al., 2015; Spotswood et al., 2015). We rely on Kurz et al., (2015) study to examine individual and structural factors of 5G technology. There are several configurations of SPT that exist (Higginson et al., 2015; Southerton, 2003; Warde, 2005). However, mostly SPT methods use practice as a unit of analysis. The practice is a routine behavior, which is composed of many interrelated elements: the form of psychological and physical activity uses of things, the background, emotional, and motivation knowledge. The practice is a block, which depends on multiple elements and specific interrelation, and cannot be simplified to a single element. The version most suitable for behavior change is the three-element model due to its simplified approach. According to Shove et al., (2012), the three-factor model consists of Material, competence, and Meanings factors. As a result, this study presented a new model (shown in Fig. 1) by combining TAM models with social practise theory elements (SPT). Based on the study background TAM and SPT factors are discussed below:

### Perceived ease of use (PEOU)

PEOU is an essential and repetitive factor proposed in TAM and has been used widely in accepting technology (Hamidi & Chavoshi, 2018; Shah et al., 2021). PEOU indicates how little effort is expected to be necessary to use a system (Davis, 1989). It is an individual's belief in eradicating physical and mental stress in a specific area. Joo et al., (2014) revealed that it is a student’s belief of using technology without any difficulties. In this study, PEOU is related to the easy access, use, interface, and flexibility of 5G smart learning technology. It is the degree of effortlessness associated with using 5G technology. In the initial phases of practising technology, simplicity of use is essential as the expectation of effort is considered necessary for using the technology (Wang et al., 2016). Moreover, users will find easy technology use more beneficial (Huang & Hsieh, 2012). Therefore we assume that:

H1: PEOU of 5G technology for smart-learning is positively related to the student’s intention (UI).

### Perceived usefulness (PU)

PU is another key factor derived from TAM and is used in new technologies acceptance models (Hamidi & Chavoshi, 2018). According to Davis (1989) and Trikoilis (2021), the assumption that using a specific technology would improve performance is referred to as PU. Althunibat (2015) also explained PU as the degree to which a system is personally dependent on improving technology performance in a defined sphere. In a smart learning context, PU signifies a certain level of confidence that smart learning will lead to improved student performance (Hao et al., 2017; Uwantege et al., 2021). Joo et al., (2014) also defined PU as the belief that technology will help to achieve student’s educational objectives. Collaboration with teachers and peers will improve efficiency in completing certain tasks. Sabah (2016). Students will embrace technology if they believe that 5G technology will improve their learning performance (Ali & Arshad, 2016; Chai et al., 2021). According to the previous research, PU has a positive influence on adoption intention. Therefore:

H2: PU of 5G technology for Smart-learning is positively related to the student’s intention (UI).

### Behavioral intention (BI)

BI defines the intentions of a user to perform a particular behavior (Davis, 1989). Studies have proven that these intentions are highly correlated with acceptance and use (Hassanzadeh et al., 2012; Mohammadi, 2015; Shah & Zhongjun, 2021). Besides, most theories in the field of technology adoption use behavioral intention as a prerequisite for user acceptance (e.g. TRA, TPB, and UTAUT). Behavioral intention (BI) is considered the core structure of TAM that predicts student’s smart learning acceptance.

### The material access (MAA) factors

"Material" is an essential part of the practice. In practice the material elements are the infrastructure, objects, hardware etc. Materials can be seen as spanning spaces of individual and social opportunity, as the availability of certain devices may explain the differences in technology usage behaviour. Changes in behaviour are linked to technological advancement. In Social practice theory, "things" are not only discourses of symbolic meaning or identity (Shove & Pantzar, 2005; Warde, 2005), but also directly participate in the behavior and reproduction of everyday life (Shove & Pantzar, 2005). In the current study, the 5G technology quality content contains rich and continuously updated learning content and facilitating conditions for smart learning practice (Almaiah et al., 2016). 5G technology will help in delivering content that is highly abstract and difficult to reconstruct. With the help of 3D virtualization technology, students will gain a profound understanding of reading. It will produce computer-generated images same to real-world content. This technology will integrate simulation and animation into education to provide the most challenging educational content better. Similarly facilitating conditions mean the existence of organizational as well as the infrastructure to back the system (Venkatesh et al., 2003). In the field of IT, It includes knowledge, resources, and support personnel. Users may not be able to practice 5G for smart learning in the absence of these conditions (Iqbal & Qureshi, 2012; Shove & Pantzar, 2005). Compared with traditional learning, smart learning is a new concept. Therefore, its execution needs users who have an understanding of the applications and services. Base on this, we hypothesis that;

H3a: The Material Access (MAA) to 5G technology for smart learning is positively related to Perceived Usefulness (PU).

H3b: The Material Access (MAA) to 5G technology for smart learning is positively related to the Perceived Ease of Use (PEOU).

### The meaning access (MEA) factors

'Meanings' are primarily based on Bourdieu (1984) concept of habitus, which proposes that a group's perception of importance is shared, thereby unifying the group. Meaning is specific to an act or thing. Shove et al., (2012) explains that this theory stresses tacit and unconscious forms of experience and knowledge, through which a collective form of understanding is established. In the context of 5G technology for smart learning, these are the individual beliefs on learning concerning the classmates, teachers, and parents etc. In general, people may think about how people close to them view their practice of technology. This inspiration may come from academic personnel or people with high social status. Further, people make self-sacrificing purchases for the benefit of others or society. The covid-19 pandemic is an unprecedented situation, which has a great significance for understanding consumer moral decision-making during and after a long-term epidemic. These environments provide users with an opportunity to imitate the fundamental meaning of consumption and its effect on themselves, society, and the environment. The covid-19 epidemic surprised consumers that their basic needs might not be completed because they may not have access to food and basic needs. At the same time, it has changed the consumer's view on how to meet social and personal needs. In terms of 5G technology consumers will consciously consider consumption and will make adoption decisions to be responsible to themselves and society. There may be a significant shift towards responsibility and pro-social consumption. Based on this solid empirical support, we assume:

H4a: The Meaning Access (MEA) to 5G technology for smart learning is positively related to Perceived Usefulness (PU).

H4b: The Meaning Access (MEA) to 5G technology for smart learning is positively related to the Perceived Ease of Use (PEOU).

### The Competency (COA) Factors

Competences mean "embodied knowledge," which originates from the studies of Bourdieu (1984) and Shilling (1991). Shove et al., (2012) define Competencies as understanding and knowledge to signify the type of experience necessary to practice successfully. Practice constitutes a "block," and its existence must depend on reality and existence. The specific interdependence between these elements cannot be condensed to any of these unique elements (Reckwitz, 2002). The competence part of practice may relate to the social-psychological theories. In the context of 5G technology for smart learning, it means the desire of an individual to accept and use new technology or risk-taking and attempts to practice new technologies (Hao et al., 2017). Highly innovative people are more willing to give a positive response to new technologies (Milošević et al., 2015). Besides, those who learn technology, are more likely innovative than others (Joo et al., 2014; Milošević et al., 2015). Kim et al., (2017) stated that such people are more concerned with attaining information about using novel technologies. Students with innovative behavior want to assent the risk of using 5G technology and are more inclined to use it. Similarly, some people think that when innovative technology is deemed to be compatible with work practice, they may realize the practicability of technology (Chau & Hu, 2001; Moore, 1991). In the context of Smart-learning through 5G, increased competencies access will positively impact as new 5G technologies will be compatible with existing technologies. Based on these arguments, we assume that:

H5a: The Competency Access (COA) to 5G technology for smart learning is positively related to Perceived Usefulness (PU).

H5b: Competency Access (COA) to 5G technology smart learning is positively related to the Perceived Ease of Use (PEOU).

## Methodology

The quantitative techniques used in this study produces results through systematic and empirical analysis of the obtained statistical data. To accomplish this, a questionnaire was prepared and sent to students in both paper and electronic form. The distribution of the questionnaires was random among groups and social networks at different universities in Beijing. Beijing was also affected during the Covid-19 epidemic, and all universities closed their academic activities. Many students are still caring their activities online. So they are well aware of the importance of smart learning through advanced network technology. All items were settled in English and Chinese depending on the respondent’s characteristics.

### Development of questionnaire

The questionnaire of this study consisted of five questions about demographic information and 24 queries about smart-learning influencing factors (MAA, COA, MEA, PEOU, PU, and BI). These items were selected from the technology adoption literature. The TAM (Davis, 1989) is used as a base theoretical model here. Hence the factors PU and PEOU are adopted from it to know the experiences of consumers' willingness to use 5G technology for learning. Material access (MAA) items are adapted from Almaiah et al., (2016) and Cho et al., (2009). The measurement items of the Competency access (COA) are adjusted from Chau and Hu (2001) and Hao et al., (2017). Similarly, the measurement items of the Meaning access (MEA) are derived from Venkatesh et al. (2003) and Du et al., (2015). For the above measures, the five-point Likert scale (1 = strongly disagree to 5 = strongly agree) was adopted. The survey was conducted in Chinese to cater to local environments. To verify the items, a pilot survey was undertaken.

### Sampling

We examined 4G and 5G users to verify the hypothesis. Whether in electronic or paper forms, the questionnaire was optional. In addition, people were asked to use the snowball method to share questionnaires with classmates and friends. To maintain anonymity, all respondents were notified that their responses would be kept anonymous and used solely for academic purposes. To represent our proposed model design, the questionnaire was divided into demographic details and measuring parameters. From September 2020 to November 2020, through an online questionnaire website http://www.sojump.com, a formal survey was conducted online. Finally, 375 valid responses were selected. Table 1 illustrates the demographic details of these respondents. The study results showed that both men (54.9%) and women (45.06%) were involved almost equally, with more than 71% of participants aged 20 to 40. About 29.06% of respondents have higher education and 62.9% have a university degree. In terms of user experience and similarity, both 4G (56.5%) and 5G (43.4%) users were examined.

**Table 1:** Demographics of the students (N = 375)

Measure	Categories	Frequency	Percentage %
Location	Beijing	243	64.8
	Shenzhen	3	0.80
	Shanghai	8	2.13
	Other	121	32.2
Gender	Male	206	54.9
	Female	169	45.06
Age	Below 20	66	17.6
	More than 20	148	39.4
	More than 30	120	32.0
	More than 40	41	10.9
Education	Other	19	5.06
	Primary level	11	2.93
	Bachelor level	236	62.9
	Master or above	109	29.06
User	4G	212	56.5
	5G	163	43.4

## Data analysis and results

The structural equation model (SEM) was applied to analyze the proposed model. SEM is a statistical models series describing the relationship of the variables (Hair Jr et al., 2016). It can express complex variable relations and provide a comprehensive representation of the model (Gefen and Straub, 2005). PLS is a technique for prediction analysis and is more appropriate for theoretical development (Urbach & Ahlemann, 2010; Shah et al., 2021). It is applied to both simple and complex models to determine the values of the variable for forecasting (Chin, 1998; Urbach & Ahlemann, 2010). Therefore this study also used PLS-SEM to test the model's complete structure. PLS technique is usually divided into two steps: first, to evaluate the model's effectiveness and reliability. Second, to evaluate the path model and determine the model's ability to assess the structural model (Hair Jr et al., 2016; Shah & Zhongjun, 2021). Also, the bootstrap method through smartpls 3.0 has been used in this study to check the path coefficients and loading factors. The following sections discuss the outcomes of these two stages.

### PLS outer model measurement results

**Fig. 2:**
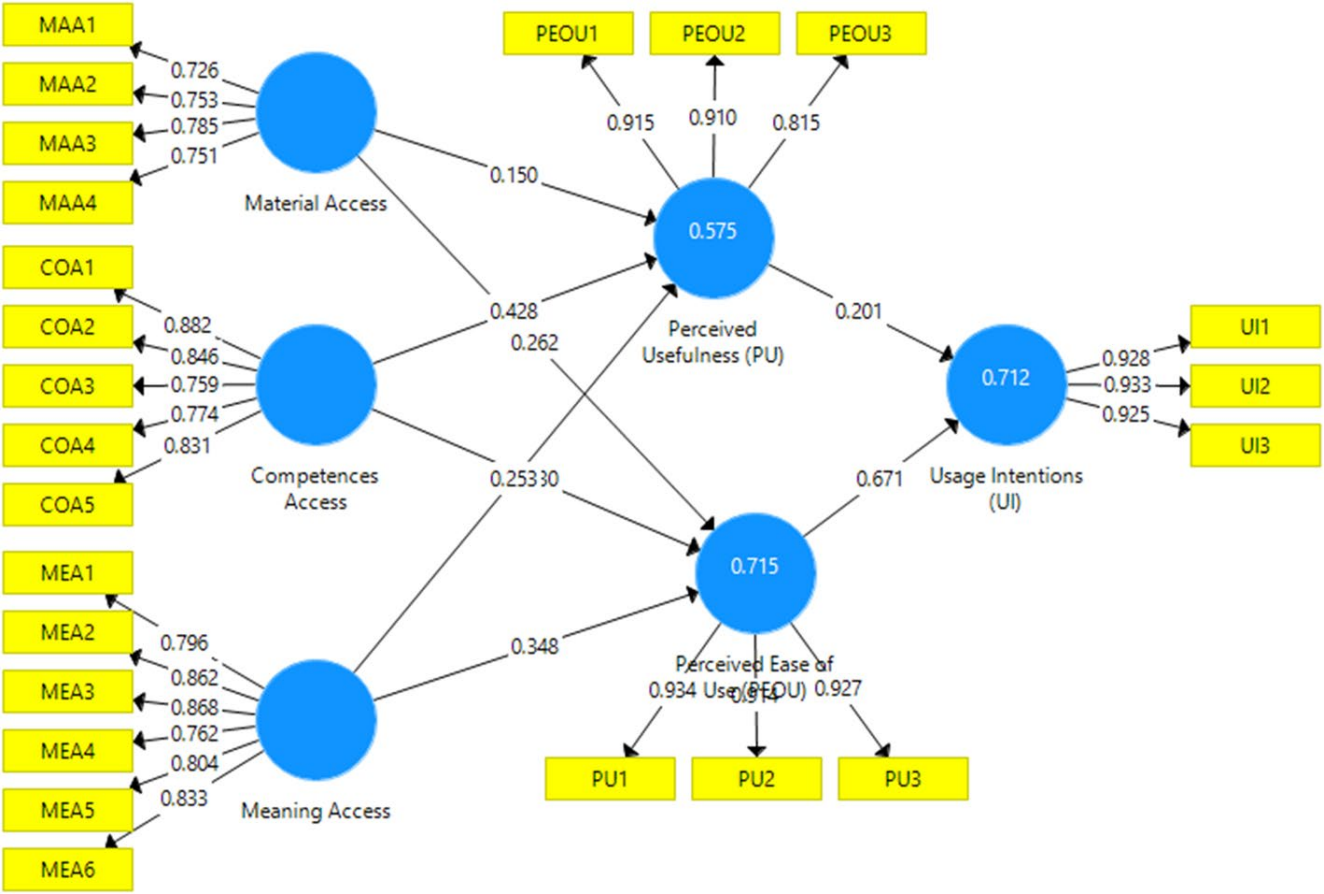
PLS algorithm estimates (Loading and Coefficients)

The validity and reliability of an external model's can be evaluated through the items' reliability, convergent, and discriminant validity. Reliability is the degree to which an item set designated for a specified construct measure a similar construct, stay reliable in distinct situations. The term validity means how to fit the instrument chosen items of a given construct are realistic. Reliability can be measure through Cronbach's alpha, composite reliability (CR) and factor loadings, convergent validity is through Average Variance Extracted (AVE), while discriminant validity is through the square root of AVE. According to Hair Jr et al. (2016) indicators, outer loading values greater than 0.6 should be retained, while the rest should be removed to increase the AVE or CR values. A total of 24 items were verified, as presented in Table 2 and Fig. 2. Cross-loadings were examined to check the items discriminant validity (Hair Jr et al., 2016). As the cross-loadings amongst constructs were more significant than the determined critical point, it verified the discriminant validity.

**Table 2:** Outer loadings

Items	Mean	Standard Deviation	Factor loading values
MAA1: As far as I know, Smart-learning via 5G can provide text, audio and 3D video content	0.717	0.605	0.726
MAA2: As far as I know, Smart-learning via 5G can provide enriched updated and animated content	0.750	0.668	0.753
MAA3: As far as I know, I have the necessary resource required for smart-learning usage	0.788	0.709	0.785
MAA4: As far as I know, The appropriate ICT infrastructure is available for smart-learning usage	0.754	0.677	0.751
COA1: If I know about new information technology, I'd like to try it somehow	0.879	0.023	0.882
COA2: I'd like to be the first to use the services, functions and applications of smart learning devices	0.843	0.025	0.846
COA3: As far as I know, Smart-learning with 5G will be flexible and can help my major study	0.755	0.057	0.759
COA4: As far as I know, Using 5G smart-learning devices will be compatible with all aspects of my work	0.770	0.055	0.774
COA5: As far as I know, 5G devices for smart-learning will be more compatible compared with other devices	0.824	0.037	0.831
MEA1: As far as I know, I would like to adopt 5G for smart learning if my instructors encourage me to do so	0.794	0.663	0.796
MEA2: As far as I know, I would like to adopt 5G for smart learning if my family encourages me to do so	0.857	0.786	0.862
MEA3: As far as I know, I would like to adopt 5G for smart learning if my peer group does	0.865	0.808	0.868
MEA4: I consider the potential impact of my actions on society and the environment	0.741	0.568	0.762
MEA5: It is important to me that the products I use do not harm society	0.788	0.653	0.804
MEA6: I would describe myself as socially responsible	0.819	0.695	0.833
PU1: As far as I know, Using 5G for smart learning can be useful for my learning	0.932	0.903	0.915
PU2: As far as I know, Using 5G for smart learning would enable me to accomplish learning tasks more quickly	0.912	0.867	0.910
PU3: As far as I know, Using 5G for smart learning will connect learners to people, content, and resources	0.925	0.889	0.815
PEOU1: As far as I know, 5G for smart learning will be easy and can use anywhere	0.913	0.883	0.934
PEOU2: As far as I know, Interact with 5G for smart-learning will be clear and understandable for me	0.909	0.879	0.914
PEOU3: As far as I know, Using 5G for smart learning may not require much effort for me	0.809	0.703	0.927
UI1: As far as I know, I intend to use 5G for smart learning	0.926	0.895	0.928
UI2: As far as I know, I'll use 5G for smart learning in the future	0.930	0.900	0.933
UI3: As far as I know, Using 5G for smart learning will motivate other learners	0.923	0.888	0.925

N = 375 (MAA = item 1,2,3,4 are of Material Access, COA = items 1, 2, 3, 4, 5 are of competences Access, MEA = items 1, 2, 3, 4, 5, 6 are of Meaning Access, PU = items 1, 2, 3, 4, are of Perceived Usefulness, PEOU = items 1, 2, 3, 4 are of Perceived Ease of use and UI = items 1, 2, 3 are of Usage Intentions)

Construct validity (CR) is another technique to assess the outer model. It determines that these processes are essential tools for expressing and measuring the investigative constructs (Hair Jr et al., 2016; Gefen and Straub, 2005). Further Convergence validity is the degree that relates or converges measures of a similar construct (Hair Jr et al., 2016). When the explained AVE's value is equivalent to or exceeds 0.5, convergence's effectiveness is verified (Fornell & Bookstein, 1982). The AVE scores of all constructs were more than 0.5, which justifies the convergent validity (Table 3). It can also be inspected through the CR of the constructs (Fornell & Bookstein, 1982). By surpassing the 0.60 threshold value, all constructs verified the composite reliability (Hair Jr et al., 2016). When calculating Cronbach's alpha to determine internal consistency, the reliability assessments should be more than 0.70 (Field, 2009). As mentioned in Table 3, all the Cronbach's alpha values surpassing the 0.70 thresholds recommended by Field (2009), Hair Jr et al. (2016) and Shah and Zhongjun (2021), thereby achieving the convergent validity second condition. Generally, the model is suitable and effective for inspecting the significance of the paths associated with these variables.

**Table 3:** Construct validity and reliability

	Cronbach’s Alpha	rho_A	Composite reliability	Average variance extracted (AVE)
Competency Access (COA) Factor	0.878	0.892	0.911	0.672
Material Access (MAA) Factor	0.756	0.772	0.840	0.568
Meaning Access (MEA) Factor	0.904	0.910	0.926	0.675
Perceived Ease of Use (PEOU)	0.915	0.916	0.947	0.855
Perceived Usefulness (PU)	0.856	0.878	0.912	0.776
Usage Intentions (UI)	0.920	0.921	0.949	0.862

Discriminant validity also examines how distinct the latent variable is from other factors (Hair Jr et al., 2016; Shah & Zhongjun, 2021). Examining the correlation matrix between constructs was one popular approach for determining discriminant validity. Especially, each prospective construct's AVE should be higher than its topmost square correlation with any other potential construct (Hair Jr et al., 2016). Table 4 demonstrates discriminant validity since all constructs in the proposed model fulfil this requirement because no off-diagonal element surpasses the diagonal element.

**Table 4:** Discriminant validity

	Competences access	Material access	Meaning access	Perceived ease of use (PEOU)	Perceived usefulness (PU)	Usage intentions (UI)
Competences access	0.820					
Material access	0.735	0.754				
Meaning access	0.747	0.640	0.822			
Perceived ease of use (PEOU)	0.783	0.728	0.762	0.925		
Perceived usefulness (PU)	0.727	0.627	0.669	0.820	0.881	
Usage intentions (UI)	0.771	0.658	0.757	0.836	0.751	0.928

### PLS inner model measurement results

**Fig. 3:**
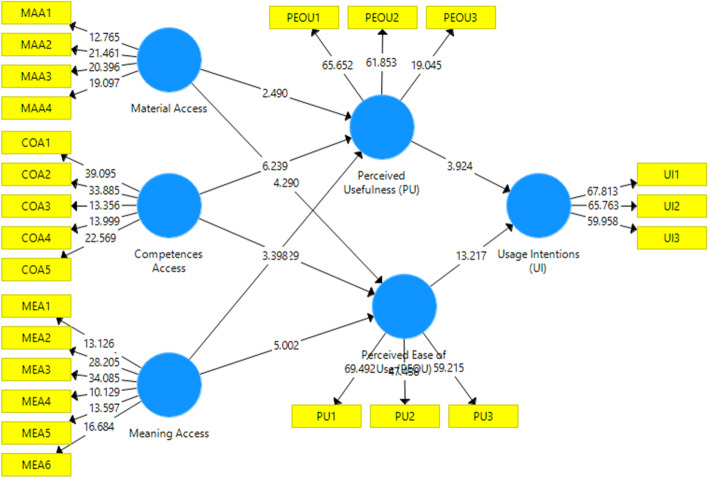
PLS bootstrapping of the model

The PLS method evaluated the structural model to determine the path relevance and predictive effect and then used the bootstrap procedure to determine the path coefficients' significance level by evaluating standard errors, confidence intervals, and T statistics (Hair Jr et al., 2016). Table 5 highlights the study hypothesis and displays the path coefficient of the latent variable as well as the critical bootstrap ratio. The bootstrap T statistic defines the estimate's stability. A 95% confidence interval above 1.96 is considered acceptable (Hair Jr et al., 2016). In the proposed model, all research hypotheses were supported. The next part will explicate the outcomes of each path. Figure 3 also illustrates the tested and verified proposed model on SmartPLS3.0 software. These conclusions offer the foundation for discussion.

**Table 5:** Path coefficient of the model

	Original sample	Sample mean	Standard deviation	T statistics	*P* values	
Competences Access → Perceived Ease of Use (PEOU)	0.330	0.324	0.062	5.329	0.000	Supported
Competences Access → Perceived Usefulness (PU)	0.428	0.421	0.069	6.239	0.000	Supported
Material Access → Perceived Ease of Use (PEOU)	0.262	0.274	0.061	4.290	0.000	Supported
Material Access → Perceived Usefulness (PU)	0.150	0.162	0.060	2.490	0.013	Supported
Meaning Access → Perceived Ease of Use (PEOU)	0.348	0.338	0.070	5.002	0.000	Supported
Meaning Access → Perceived Usefulness (PU)	0.253	0.244	0.075	3.398	0.001	Supported
Perceived Ease of Use (PEOU) → Usage Intentions (UI)	0.671	0.663	0.051	13.217	0.000	Supported
Perceived Usefulness (PU) → Usage Intentions (UI)	0.201	0.204	0.051	3.924	0.000	Supported

## Discussion

This study aims to explore the adoption of smart learning through 5G in educational institutions in or after the Covid-19. We categorized and tested these factors by hypothesis. These characteristics have been identified as major determinants of technology acceptance in much previous research, but less emphasis has been paid to the implications of these characteristics on smart learning acceptability. Each of the stated factors has been further reviewed and discussed here.

As a result of the path analysis, all the hypotheses are supported. The material access factors MAA significantly influenced PU&PEOU (MAA → PU, MAA → PEOU) which was also supported by Almaiah et al., (2016) and Cheng (2012). It suggests that if learners perceived smart learning material content as up to date and complete, they will feel it more valuable. The developers and education professionals should be aware that smart learning devices and content must be appropriate and personalized based on student’s needs, which will surge their usefulness. Further, students may also see that they should have minimum requirements for using these technologies, and improving these conditions will significantly impact intentions to use. The outcomes of the study further backing the hypothesis ((COA → PU, COA → PEOU) that is competency access (COA) factor help in adoption intentions. This makes it easy for users to perceive that it is not complicated for them and is related to their work to use this technology. Students have adequate knowledge and capability to use new technologies. In addition, even if the usage is complex, they can easily find ways to solve it. Further, the hypotheses associated with Meanings (MEA) access factors ((MEA → PU, MEA → PEOU) support the argument that user social consumption can influence the PU, culturally, socially, politically and so on. Before using 5G smart learning technology users will think that his/her consumption will not affect society. This prosaic consumption belief is considered worthwhile. As the whole sample of this study is students, mostly they have the understanding to analyze the worth of smart-learning technology so they don’t need an endorsement from others. Based on these results, it can be determined that society's belief may be effective on PU and PEOU. This is because 5G technology for smart learning is a societal need, especially after Covide-19.

As the SPT is a natural doorway for interdisciplinary thinking, which is necessary when behavioral issues have a vast scale and complicated foundations, using it for the study of smart learning practise contributes significantly to behavior modification (Marsden et al., 2014). SPT can give a thorough enough study of the problem to present ‘‘a wide variety of modification options (Rettie et al., 2012). This wide variety of options is founded on the idea that altering a practise necessitates breaking or questioning the bonds that bind its numerous interconnected parts together (Shove & Pantzar, 2005). From this little sample conversation, it is clear that to effect change, a plethora of linkages between connected materials, meanings and competencies must be addressed. As a result, the architecture of smart learning practise may necessitate a variety of integrated law, infrastructure, policy, and marketing initiatives.

## Conclusion, limitations and future works

This study varies from other studies on the adoption of 5G for smart learning in educational institutions. It presents a more inclusive model based on China's current needs and social conditions, which proposes that the choice of 5G for smart learning depends on a combination of certain factors (Materials, Meanings, and competencies). The outcomes of this study were conducted in different universities in China. Regarding the material meaning and competency characteristics as part of the premise of student’s beliefs. It was shown that these factors have an important impact on smart learning via 5G adoption. This study provides a priori smart learning acceptance by providing valuable information about key characteristics that support students' perceptions and beliefs of enhancing students' BI to adopt 5G technology for smart learning. The study has some limitations too. Even though we employed marker variables to look for common method bias, our findings are based on cross-sectional data. This study only covers a few Chinese universities, therefore the results can only be generalized to Chinese universities. Thus universities of different regions having different characteristics in terms of psychology, education, and demographics should be considered in future studies. Future studies should be conducted to develop adoption models for smart learning based on the other characteristics identified as prerequisites for smart learning acceptance. Although this study analyses the feasibility and substance of an interdisciplinary approach briefly, it is noted that it would be best conceptualised through the future development of a set of SPT-based tools. As a result, while this article is only the beginning of a new approach to behaviour, it is hoped that it will contribute to a rich new gusset of analysis that will illustrate the issue of smart learning through 5G technology, allowing the development of effective, practical exposure to smart learning behavioural changes.

## Data Availability

The data that support the findings of this study are available from the corresponding author upon reasonable request.

## Abbreviations

TAM: Technology Acceptance Model
SPT: Social Practice Theory
SEM: Structural Equation Modeling
MAA: Material Access
COA: Competency access
MEA: Meanings Access
UTAT: Unified Theory of Acceptance of Technology
TPB: Theory of Planned Behavior
TRA: Theory of Reasoned Actions
PEOU: Perceived Ease of Use
PU: Perceived Usefulness
UI: Student’s Intention
BI: Behavioral intention
CR: Composite Reliability
AVE: Average Variance Extracted
